# Chronic Neurophysiological Effects of Repeated Head Trauma in Retired Australian Male Sport Athletes

**DOI:** 10.3389/fneur.2021.633320

**Published:** 2021-03-09

**Authors:** Alan J. Pearce, Dawson J. Kidgell, Mark A. Tommerdahl, Ashlyn K. Frazer, Billymo Rist, Rowena Mobbs, Jennifer Batchelor, Michael E. Buckland

**Affiliations:** ^1^College of Science, Health and Engineering, La Trobe University, Melbourne, VIC, Australia; ^2^Department of Physiotherapy, Faculty of Medicine Nursing and Health Science, Monash University, Melbourne, VIC, Australia; ^3^Department of Biomedical Engineering, University of North Carolina, Chappell Hill, NC, United States; ^4^Cortical Metrics, Carrboro, NC, United States; ^5^Department of Neurology, Macquarie University Hospital, Macquarie University, Sydney, NSW, Australia; ^6^Department of Psychology, Macquarie University, Sydney, NSW, Australia; ^7^Department of Neuropathology, Royal Prince Alfred Hospital, Sydney, NSW, Australia; ^8^Brain and Mind Centre, University Sydney, Camperdown, NSW, Australia

**Keywords:** concussion, sports, transcranial magnetic stimulation, somatosensory, mental fatigue, motor cortex

## Abstract

**Aim:** This study investigated the somatosensory and corticomotor physiology of retired contact sport athletes with a history of repeated concussion/subconcussion head trauma.

**Methods:** Retired male athletes with a history of playing contact sports and repeated head trauma (*n* = 122) were divided into two groups: those who expressed concerns regarding their mental and cognitive health (“symptomatic”: *n* = 83), and those who did not express any ongoing concerns (“asymptomatic”: *n* = 39). Both groups were compared to age-matched male controls (*n* = 50) with no history of concussions or participation in contact sports, an absence of self-reported cognitive, or mood impairments. Transcranial magnetic stimulation (TMS) and vibrotactile stimulation were used to assess corticomotor and somatosensory pathways respectively. TMS and vibrotactile stimulation were correlated to self-reported responses using the Fatigue and Related Symptom Survey. Linear regression was used to associate concussion history with TMS, somatosensory variables.

**Results:** Significant differences were found in symptom survey scores between all groups (*p* < 0.001). TMS showed significant differences between the “symptomatic” and control groups for intracortical inhibition and paired pulse TMS measures. Somatosensory measures showed significant differences for reaction time (*p* < 0.01) and reaction time variability (*p* < 0.01) between the “symptomatic” group to the “asymptomatic” and control groups. For other somatosensory measures, the “symptomatic” measures showed differences to the “control” group. Correlations showed significant associations between severity of symptom reporting with TMS and somatosensory measure, and regression revealed the number of concussions reported was shown to have significant relationships to increased intracortical inhibition and poorer somatosensory performance.

**Conclusion:** This study shows that retired contact sport athletes expressing chronic symptoms showed significant pathophysiology compared to those with no ongoing concerns and non-concussed controls. Further, there is a linear dose-response relationship between number of reported concussions and abnormal neurophysiology. Neurophysiological assessments such as TMS and somatosensory measures represent useful and objective biomarkers to assess cortical impairments and progression of neuropsychological impairment in individuals with a history of repeated head trauma.

## Introduction

International attention toward understanding the relationship between repetitive head trauma experienced in contact sports and the increased risk of chronic neurological impairment later in life continues to grow. Recent epidemiological and cohort studies suggest that increased exposure increases the risk and prevalence for a range of neurological impairments and neurodegenerative diseases later in life (1, 2).

While the majority of research into long-term neurological impairments following a history of head trauma has been generated from North America, particularly from boxing and American football, there is emerging data from other countries, and in other contact sports including ice hockey, association football (soccer), rugby union and league, and Australian football (3–6). In Australia, anecdotal reports of increasing numbers of older retired athletes expressing personal concerns regarding cognitive and mental health [e.g., (7, 8)], has reinforced previous studies reporting concerning findings. For example, a preliminary study in retired rugby league players (*n* = 16) using magnetic resonance spectroscopy showed changes in grey matter glutathione, and functional measures of manual dexterity in their non-dominant hand, but no changes on measures of cognition, depression or anxiety compared to age- and education-matched controls (9). More recent work in recently retired professional rugby league players (*n* = 11) using diffusion tensor imaging showed significant alterations in long fibre tracts, including corticospinal tracts, despite no differences in measures of depression, anxiety, stress, or post-concussion symptoms compared to age and education-match controls (10).

Studies in Australian retired athlete cohorts (4, 11) have also been undertaken using transcranial magnetic stimulation (TMS), which has supported work in Canadian and New Zealand retired athletes (12, 13). A non-invasive technique, TMS allows for the identification of changes in specific, functionally relevant corticomotor circuits within healthy adults, but can also detect pathophysiology of repeated brain trauma (14, 15), specifically γ-aminobutyric acid (GABA) intracortical inhibition. Studies of Australian football (*n* = 40) and rugby league (*n* = 25), where retired athlete groups were compared to age- and education-matched controls, TMS showed changes in GABA type A (GABA_A_) and type B (GABA_B_) receptor activity that were associated with differences in fine motor control, reaction time, working memory, flexibility of attention, and associative learning (4, 11). However, to date TMS studies focusing on long-term outcomes have yet to include a retired athlete's group with no self-reported concerns about depression, anxiety, memory, fatigue, or stress, despite a similar history of participation in contact sports and concussions.

Similar to TMS, understanding the physiology of somatosensory information processing can provide insights into cognitive performance following a history of repetitive brain trauma. It has been described that cognitive performance is relatively stable in those who have suffered mild traumatic brain injury (mTBI). For example, Stenberg et al. (16) reported in mTBI patients who despite self-reporting cognitive impairments, showed stable cognitive performance post injury. However, it has been argued that established cognitive testing may not be rigorous enough to detect transient impairment, or undefined compensatory neural mechanisms may allow for the maintenance of cortical information processing despite evidence of anatomical or physiological decline (17, 18).

Utilising somatosensory measures can address this issue by quantifying various aspects of a participant's cognitive information processing capacity (18, 19). A method to objectively quantify somatosensory processing is via vibrotactile processing. This technique does not simply reflect alterations in tactile perception, but rather differences in cortical information processing capacity mediated by GABAergic and/or NMDA receptor activity (20–22). Vibrotactile assessment has previously demonstrated central information processing alterations following acute concussion (22), as well as in those with persistent post-concussion symptoms (23, 24). However, somatosensory studies have not yet been conducted in older athletic cohorts with a history of repeated head trauma, decades after their last reported concussion and well beyond the typical duration of persistent post-concussion symptoms.

This study reports on TMS and somatosensory findings in retired contact sport athletes with self-reported concerns about impaired cognition and mood (symptomatic). A novel aspect to this study was comparison of symptomatic athletes to a group of retired athletes with no ongoing concerns (asymptomatic), with both groups compared to age- and education-matched control group. We hypothesised that the symptomatic group would show abnormal intracortical inhibition, compared to the asymptomatic and control groups.

## Materials and Methods

### Participants

Retired male athletes (*n* = 122) across a range of sports including Australian rules football (*n* = 77), rugby league (*n* = 33), rugby union (*n* = 5), boxing (*n* = 4), and car racing (*n* = 3) were independently recruited for the study. All participants were screened and excluded if reporting serious neurological disease (e.g., idiopathic generalized epilepsy, central nervous system neoplasm, brain tumour or neurodegenerative disease), sleep disorders (i.e., sleep apnea), or history of psychiatric disorders (e.g., personality disorders, schizophrenia spectrum disorder, bipolar and related disorders, addictive behaviour disorder) as well as history of learning disabilities, or brain injury unrelated to contact sports. The retired playing group was divided into those who expressed concerns regarding their mental and cognitive health as a result of their history of repetitive head trauma in sport (“symptomatic”: *n* = 83), and those who acknowledged they had a history of head trauma from sport, but did not express any ongoing concerns (“asymptomatic”: *n* = 39). Both groups were compared to age-matched male controls (*n* = 50) with no history of concussions or participation in contact sports, an absence of self-reported cognitive or behavioural concerns, and who had no diagnosed neurological impairments or psychiatric disorders.

All participants provided written informed consent to participate in the study. The Institutional Review Board approved all study protocols (HEC18005) conforming to the guidelines set out by the Declaration of Helsinki.

### Study Design

This study utilised a between-groups design, with participants visiting the laboratory for a single visit. All participants completed pre-screening for suitability to TMS, symptom survey, somatosensory processing via vibrotactile stimulation and transcranial magnetic stimulation. Vibrotactial stimulation and TMS were conducted in a counterbalanced order to reduce potential stimulation serial effects.

#### Symptom Survey

Participants completed the *mental fatigue and related symptom survey* (25). The individual responds using a scale corresponding to response statements given from “0” (no concern) to “3” (maximal severity). Statements described difficulties the participant may encounter that affect their daily activities. Questions were related to aspects such as intensity, frequency and duration of difficulties. The questionnaire also allows for the participant to score between the given alternatives (0.5, 1.5, 2.5). The survey has been shown to have high reliability reporting Chronbach's alpha of 0.94 (25), and in studies of persistent post-concussion symptoms (23, 24) and preliminary investigations in older athletes (26).

#### Somatosensory Assessment

Somatosensory assessment was undertaken by using a portable vibrotactile stimulation device (Cortical Metrics, USA). The device, similar in size and shape to a standard computer mouse, contains two cylindrical probes (5 mm diameter) positioned at the top and front of the device. These probes, driven by the computer via a USB cable, provided a light vibration stimulus, at frequencies between 25 and 50 Hz that is sensed by the participant's index (D2) and middle (D3) digits of their non-dominant hand.

Participants completed the battery involving seven discrete tasks, using their dominant hand via a computer mouse, utilised in previous studies for a combined testing time of 20–25 min (18, 20, 22–24, 27, 28). For each task in the battery, a simple tracking procedure that utilized a two-alternative forced choice paradigm was used to determine an individual's difference distinguished threshold for stimulus (22). Familiarization was performed before each test for participant orientation, requiring correct responses on three consecutive trials prior to commencement of the test where data would be acquired. There was no feedback or knowledge of the results made to participants during testing (22).

Data measured included reaction time (responding as quickly as possible with the dominant hand when a single pulse stimulus was provided to the non-dominant digit D2), amplitude discrimination (deciding which digit, D2 or D3, felt a greater intensity during separate sequential vibration of the digits, and simultaneous vibration of the digits), temporal order judgment (determining which digit, D2 or D3, felt the stimulus pulse first), and duration discrimination (which of the two stimuli presented simultaneously vibrated for a longer time). For full detailed protocols the reader is referred to Holden et al. (21) and Tommerdahl et al. (22).

#### Transcranial Magnetic Stimulation and Electromyography

Applying previously described protocols (29–31), TMS was applied over the contralateral motor cortex with surface electromyography (sEMG) recording 500 ms sweeps (100 ms pre-trigger, 400 ms post-trigger; PowerLab 4/35, ADInstruments, Australia). The sEMG activity was recorded using bipolar Ag/AgCl electrodes, with an intra-electrode distance of 2 cm positioned over the first dorsal interosseous (FDI) muscle of the participant's dominant hand adhering to the Non-Invasive Assessment of Muscles (SENIAM) guidelines for sEMG (32).

TMS was delivered using a MagStim 200^2^ stimulator (Magstim, UK) using a figure of eight D70 remote coil (Magstim, UK). For reliability of coil placement participants wore a snugly fitted cap (EasyCap, Germany), positioned with reference to the nasion-inion and interaural lines. The cap was marked with sites at 1 cm spacing in a latitude-longitude matrix to ensure reliable coil position throughout the testing protocol (33).

Following identification of the “optimal site,” defined as the site with the largest observed MEP (33), active motor threshold (aMT) was determined, during a controlled, low-level voluntary contraction of the FDI muscle at 10% of Maximal Voluntary Contraction (MVC). The aMT was identified by delivering TMS stimuli (5% of stimulator output steps, and in 1% steps closer to threshold) at intensities from a level below the participant's threshold until an observable MEP of at 200 μV and associated cSP could be measured in at least five of ten stimuli (34). Stimuli were delivered in random intervals (between 6 and 10 s) at intensities of 130, 150, and 170% of aMT. Twenty stimuli were presented in random intervals of four sets of five pulses per set, with a break of 30 s provided between sets and intensity levels to reduce the possibility of muscular fatigue (31).

Single pulse active MEP latency was calculated as the time between stimulation of the motor cortex to the onset of the MEP (35). MEP amplitudes were measured from the peak-to-trough difference of the waveform. Duration of the cSP was calculated from the onset (deflection) of the MEP waveform to the return of uninterrupted EMG (36). As recently suggested by Škarabot et al. (37), the most influencing confounding factor on cSP duration is the preceding MEP; therefore to reflect a balance between excitatory and inhibitory mechanisms, we used MEP:cSP ratios to compare between groups and reduce between-participant variability (38). We have previously published MEP: cSP ratios in a cohort with persistent post-concussion symptoms (24).

Paired-pulse MEPs for short latency intracortical inhibition (SICI) and long intracortical inhibition (LICI) were measured with the FDI using an interstimulus interval (ISI) of 3 and 100 ms, respectively. SICI was undertaken with a conditioning stimulus of 80% aMT and a test stimulus of 130% aMT, while LICI was completed using a suprathreshold conditioning and test stimulus at 130% aMT; stimulation ratios and ISIs which we have previously published in post-concussion population groups (4, 11, 39). Twenty sweeps were delivered, in four sets of five, at random intervals between 8 and 10 s, with SICI expressed as a ratio of the paired-pulse MEP to the single pulse MEP measured also at 130%, and LICI expressed as a ratio of the test stimulus to the conditioning stimulus (4, 11, 39, 40).

#### Statistical Analysis

All data were screened for normal distribution using Kolmogorov-Smirnov (K-S) tests, showing data to be skewed. Following log transformation of the data, K-S tests revealed normal distribution. Further, Levene's tests for equal variances found that the assumption of homogeneity of variance was met with all variables; therefore parametric testing using log transformed data were employed with equal variances assumed. However, the mental fatigue and related symptom survey variables did not meet the criteria for the assumption of homogeneity. Consequently, Welch's test for unequal variances was applied for these variables.

All dependent variables (participant characteristics, cortical metrics and TMS) were compared using a one-way ANOVA or independent *t*-test (where appropriate). Where ANOVA detected differences, *post-hoc* testing with Tukey correction was used. Values are presented as mean (±95 CI). Correlations (Pearson's *r*) were performed between dependent variables and linear regression was performed between the number of concussions reported and dependent variables. Cohen's *d* for small (≤0.2), medium (0.21–0.8) and large (≥0.81) (41) effect size differences are presented between groups for TMS and somatosensory results. Data is presented as mean (±95%CI) and alpha was set at *p* ≤ 0.05.

## Results

All participants recruited completed the assessments with no adverse effects. Group comparisons (Table 1) showed no difference in age [*F*_(2, 169)_ = 0.78; *p* = 0.45]; or education [*F*_(2, 169)_ = 2.19, *p* = 0.11]. Between the two post-concussion groups there were no differences in time since their last reported concussion [*t*_(120)_ = 1.15 *p* = 0.25; *d* = 0.23], or the number of previously reported concussions [*t*_(38)_ = 1.57; *p* = 0.11; *d* = 0.34].

**Table 1:** Participant demographics and related symptom survey score.

	**Mean age (years)**	**Mean education (years)**	**Mean number of reported concussions**	**Mean time since last concussion (years)**	**Mean *mental fatigue and related symptom* score**
“Symptomatic” (*n =* 83)	48.5 ± 9.7	14.2 ± 1.6	6.3 ± 5.8	18.8 ± 8.1	21.2 ± 4.5^**^
“Asymptomatic” (*n =* 39)	47.6 ± 10.6	14.7 ± 1.5	4.6 ± 3.7	16.8 ± 9.2	8.7 ± 4.4n^*^
Control (*n =* 50)	48.5 ± 11.7	15.3 ± 2.7	n/a	n/a	2.0 ± 2.8

** Significant difference between “symptomatic to asymptomatic” (p < 0.001) and control (p < 0.001);

* *significant difference between “asymptomatic” to control (p < 0.001). n/a, not applicable*.

*Data reported in mean (±SD)*.

### Fatigue and Related Symptoms Scores

**Figure 1:**
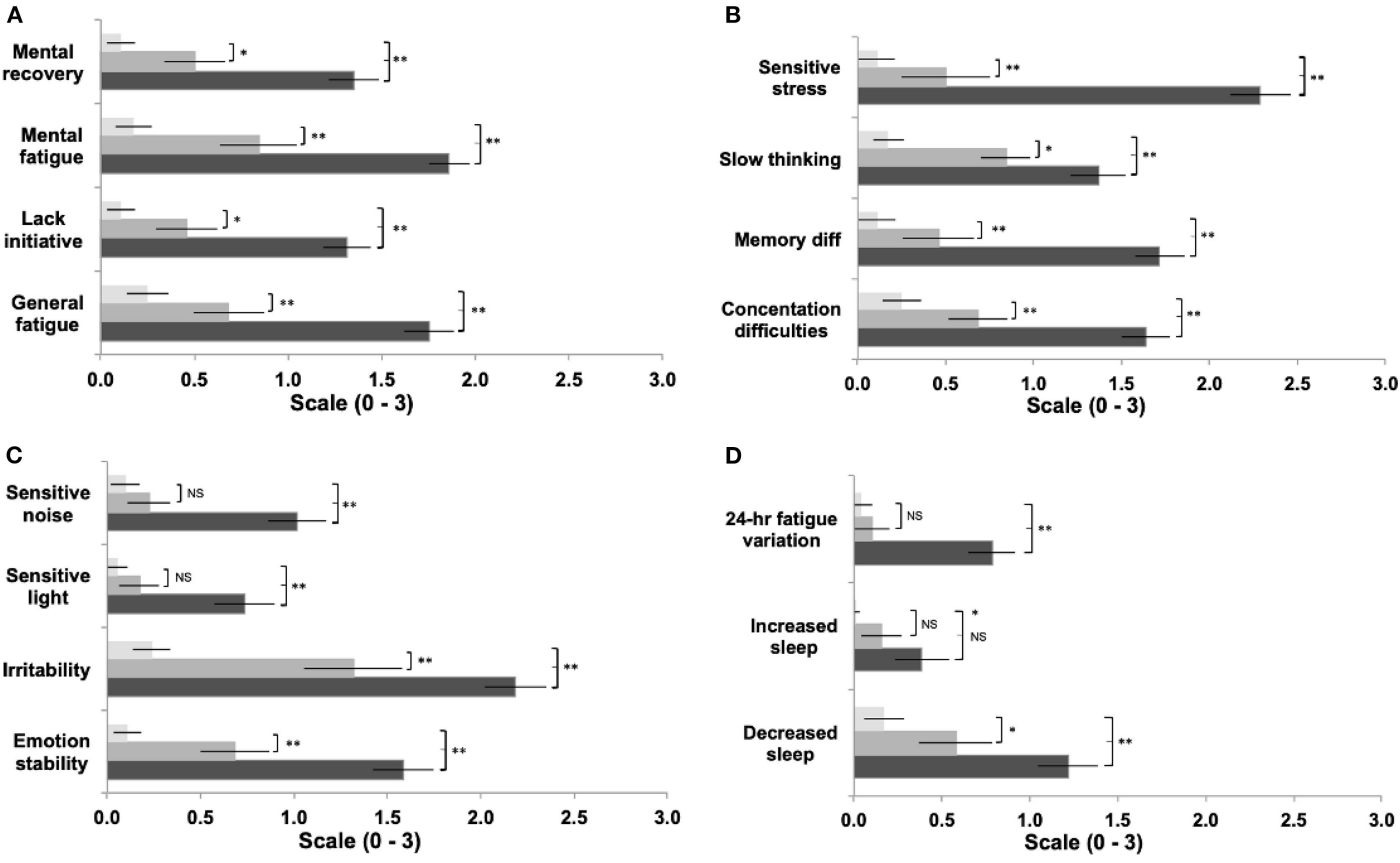
Comparison between groups of symptom items reported in the Fatigue and Related Symptom Survey **(a-d)**. Light bars represent controls, grey bars represent 'asymptomatic' group, black bars represent the 'symptomatic' group. (Data expressed and mean ± 95%CI; *p<0.05; **p<0.01).

A significant difference was found between groups for the total sum of scores for mental fatigue and related symptom questionnaire [*F*_(2, 169)_ = 369.37; *p* < 0.001]. *Post-hoc* tests showed that the “symptomatic” group reported a significantly greater overall severity score, compared to “asymptomatic” (*p* < 0.001, *d* = 5.3) and control groups (*p* < 0.001, *d* = 2.8); and significantly greater severity across all individual items (Figures 1A–D) compared to the “asymptomatic” group (items *p* < 0.001; *d*: 0.87–1.77), except *increased sleep* (*p* = 0.08 *d* = 0.42), and the control group (items *p* < 0.001; *d*: 0.94–3.97). The “asymptomatic” group showed increased severity across items compared to the control group (items *p* < 0.01; *d*: 0.76–1.67), except for *sensitivity to light* (*p* = 0.60 *d* = 0.45), *sensitivity to noise* (*p* = 0.13 *d* = 0.40), *increased sleep* (*p* = 0.15 *d* = 0.66), and *24-h variations* (*p* = 0.82 *d* = 0.24).

### Somatosensory Assessment

**Figure 2:**
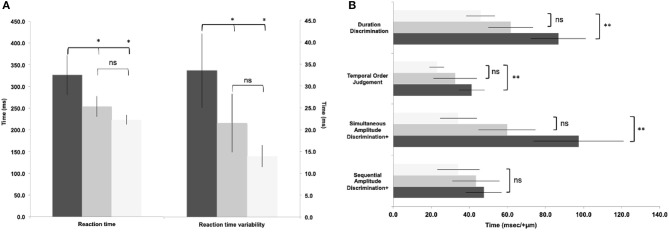
Reaction time **(a)** and somatosensory data **(b)** between groups. Light bars represent controls, grey bars represent 'asymptomatic' group, black bars represent 'symptomatic' group. (Data expressed as group mean ± 95%CI; ns - not significant; *p<0.05; **p<0.01.

One-way ANOVA showed significant differences between groups in all variables (*p* = 0.008–<0.001) except sequential amplitude discrimination [*F*_(2, 169)_ = 1.87, *p* = 0.16, Figures 2A,B]. *Post hoc* comparisons showed statistically significant slowed reaction times (Figure 2A) between “symptomatic” to “asymptomatic” and control groups (*p* = 0.004, *d* = 0.72; *p* < 0.001, *d* = 1.20, respectively). No differences were found between the “asymptomatic” and control groups (*p* = 0.15, *d* = 0.36). Similarly *post hoc* comparisons showed statistically significant greater reaction time variability (Figure 2A) between the “symptomatic” and “asymptomatic”, and control groups (*p* = 0.02, *d* = 0.56; *p* < 0.001, *d* = 1.19, respectively). No differences were seen between the “asymptomatic” and control groups (*p* = 0.22). *Post hoc* analyses for simultaneous amplitude discrimination (Figure 2B) showed no statistical difference between “symptomatic” and “asymptomatic” (*p* = 0.28, *d* = 0.68) groups. However, a significant difference reflecting the inability to discriminate in the strength of the stimuli presented, was observed between “symptomatic” and control groups (*p* = 0.002, *d* = 1.29). No statistical difference was seen between the “asymptomatic” and control groups (*p* = 0.16, *d* = 0.84). *Post hoc* comparisons for temporal order judgment (Figure 2B) showed no significant difference between “symptomatic” and “asymptomatic” groups (*p* = 0.08, *d* = 0.35), but a significant difference in the inability to detect which stimuli was presented first was seen between “symptomatic” and controls (*p* = 0.001, *d* = 1.18). Duration discrimination showed no differences, between “symptomatic” and “asymptomatic” groups (*p* = 0.09, *d* = 0.68), or “asymptomatic” and control groups (*p* = 0.07, *d* = 0.66). A significant difference was found in the inability of the “symptomatic” group to detect which stimuli was presented for a longer period, compared to the control group (*p* < 0.001; *d* = 1.28). Correlations between somatosensory data and reported symptoms are presented in Table 2.

**Table 2:** Somatosensory measures and self-reported symptoms from all participants who reported concussions (*n* = 122).

	**Reaction time**	**Reaction time variability**	**Sequential amplitude discrimination**	**Simultaneous amplitude discrimination**	**Time order judgement**	**Duration discrimination**
Overall score	0.39^***^	0.43^***^	0.17	0.46^***^	0.36^***^	0.46^***^
General fatigue	0.34^**^	0.41^***^	0.09	0.38^***^	0.36^***^	0.39^***^
Lack initiative	0.36^***^	0.43^***^	0.18	0.40^***^	0.21	0.43^***^
Mental fatigue	0.36^***^	0.41^***^	0.13	0.43^***^	0.32^**^	0.47^***^
Mental recovery	0.24^*^	0.24^*^	0.13	0.26^*^	0.27^*^	0.30^**^
Concentration difficulties	0.29^**^	0.35^**^	0.20	0.33^**^	0.28^**^	0.43^***^
Memory difficulties	0.32^**^	0.41^***^	0.15	0.40^***^	0.26^*^	0.29^*^
Slow thinking	0.33^**^	0.36^***^	0.20	0.50^***^	0.34^**^	0.49^***^
Sensitive to stress	0.24^*^	0.24^*^	0.09	0.35^**^	0.24^*^	0.42^***^
Emotional stability	0.23^*^	0.23^*^	0.09	0.41^**^	0.36^***^	0.32^**^
Irritability	0.23^*^	0.33^**^	0.12	0.30^**^	0.27^*^	0.31^**^
Sensitive to light	0.38^***^	0.30^**^	0.13	0.41^***^	0.25^*^	0.31^**^
Sensitive to noise	0.41^***^	0.34^**^	0.17	0.54^***^	0.27^*^	0.40^***^
Decreased sleep	0.18	0.26^*^	0.18	0.33^**^	0.37^***^	0.40^***^
Increased sleep	0.65^***^	0.66^***^	0.11	0.11	0.31^**^	0.12
24-h variation	0.20	0.24^*^	−0.02	0.25^*^	0.22^*^	0.27^*^

* p < 0.05;

** p < 0.01;

*** *p < 0.001*.

### Transcranial Magnetic Stimulation

No significant differences between groups were found for latency [*F*_(2, 169)_ = 2.657, *p* = 0.073] or aMT [*F*_(2, 169)_ = 1.253, *p* = 0.288). Correlations for TMS variables with self-reported symptoms are presented in Table 3 and TMS and somatosensory variables are presented in Table 4.

**Table 3:** TMS and Self-reported symptoms from all participants who reported concussions (*n* = 122).

	**MEP:cSP 130**	**MEP:cSP 150**	**MEP:cSP 170**	**SICI**	**LICI**
Overall score	−0.18^*^	−0.25^**^	−0.28^**^	0.11	0.25^***^
General fatigue	−0.07	−0.13	−0.15	−0.01	0.13
Lack initiative	−0.06	−0.21^*^	−0.24^**^	0.06	0.16^*^
Mental fatigue	−0.11	−0.17	−0.20	0.02	0.20^*^
Mental recovery	−0.10	−0.23^*^	−0.21	0.06	0.14
Concentration difficulties	−0.10	−0.20	−0.24	0.10	0.13
Memory difficulties	−0.10	−0.15	−0.22	0.09	0.19^*^
Slow thinking	−0.12	−0.22^*^	−0.27	0.11	0.19^*^
Sensitive to stress	−0.29^***^	−0.28^**^	−0.30	0.19^*^	0.31^***^
Emotional stability	−0.23^**^	−0.32^***^	−0.33	0.16^*^	0.30^***^
Irritability	−0.27^***^	−0.24^**^	−0.25	0.13	0.33^***^
Sensitive to light	−0.09	−0.18^*^	−0.21	0.07	0.22^**^
Sensitive to noise	−0.08	−0.17	−0.16	−0.08	0.22^**^
Decreased sleep	−0.11	−0.18^*^	−0.20	0.03	0.17^*^
Increased sleep	−0.02	−0.02	−0.08	0.04	0.15^*^
24–h variation	0.01	−0.11	−0.09	0.05	0.14

* p < 0.05;

** *p < 0.01*;

*** *p < 0.001*.

**Table 4:** TMS and cortical metrics data from all participants who reported concussions (*n* = 122).

	**MEP:cSP 130**	**MEP:cSP 150**	**MEP:cSP 170**	**SICI**	**LICI**
Reaction time	0.22^*^	0.20^*^	0.26^***^	−0.05	0.09
Reaction time variability	0.31^***^	0.32^***^	0.33^***^	−0.12	0.03
Sequential amplitude discrimination	−0.03	−0.04	−0.15	0.09	−0.04
Simultaneous amplitude discrimination	−0.06	−0.02	0.09	0.09	−0.05
Time order judgment	−0.08	−0.07	−0.17^*^	−0.25^***^	−0.14
Duration discrimination	0.06	0.14	0.19^*^	−0.17^*^	0.02

* p < 0.05; **p < 0.01;

*** *p < 0.001*.

#### MEP:cSP Ratio

**Figure 3:**
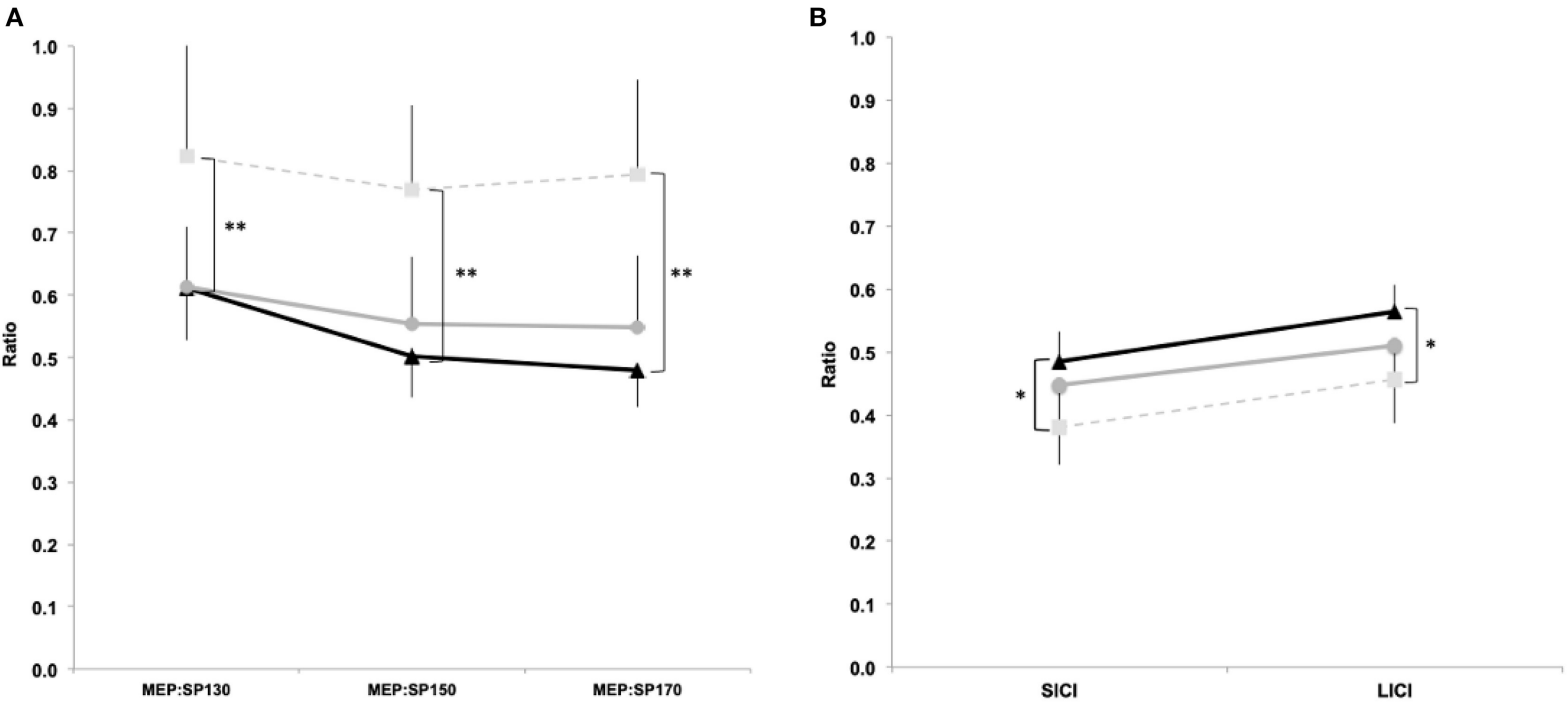
TMS single and paired pulse data. MEP:cSP ratios between groups for 130%, 150% and 170% aMT **(a)** and SICI (3 ms) and LICI (100 ms) paired pulse **(b)**. Light squares/dashed line represent controls, grey circles/solid line represent 'asymptomatic' group, and black triangles/solid line represent 'symptomatic' group. (Data expressed as group mean ± 95%CI; ns - not significant; *p<0.05; **p<0.01).

Significant differences were observed for MEP:cSP ratios at 130% [*F*_(2, 169)_ = 4.24, *p* = 0.018], 150% [*F*_(2, 169)_ = 6.87, *p* = 0.001] and 170% [*F*_(2, 169)_ = 7.42, *p* = 0.001). *Post hoc* analyses revealed significant reduction in intracortical inhibition between “symptomatic” and control groups at 130% (*p* = 0.01, *d* = 0.44), 150% (*p* = 0.001, *d* = 0.82), and 170% (*p* = 0.001, *d* = 0.77), however no significant differences were detected between “symptomatic” and “asymptomatic,” and “asymptomatic” and control groups at all stimulus intensities (Figure 3A). Significant correlations were observed at all intensities of TMS with reaction time and reaction time variability (Table 4). Correlations were also found in time order judgement and duration discrimination with the highest intensity of TMS stimulation (170% aMT; Table 4).

#### SICI and LICI Ratio

Significant differences were observed for SICI [*F*_(2, 169)_ = 4.09, *p* = 0.02], and LICI [*F*_(2, 169)_ = 4.10, *p* = 0.02]. *Post hoc* analyses showed significantly increased SICI (*p* = 0.02, *d* = 0.56) and LICI (*p* = 0.02, *d* = 0.49) between “symptomatic” and control groups. No differences were found between “symptomatic” and “asymptomatic” groups for SICI (*p* = 0.71, *d* = 0.16) and LICI (*p* = 0.54, *d* = 0.16), and between “asymptomatic” and control groups for SICI (*p* = 0.21, d = 0.35) and LICI (*p* = 0.32, *d* = 0.29; Figure 3B). A significant negative correlation was found between SICI and time order judgement (*r* = −0.25, *p* = 0.02) and duration discrimination (*r* = −0.17, *p* = 0.03; Table 4).

### Effect of Concussion History on TMS and Somatosensory Assessments

The number of concussions reported was shown to have significant relationships to Somatosensory and TMS variables. There were strong positive linear relationships between the number of reported concussions with reaction time [*r* = 0.30, *r*^2^= 0.09, *F*_(1, 160)_ =7.73, *p* = 0.007], reaction time variability [*r* = 0.36, *r*^2^= 0.13, *F*_(1, 160)_ = 11.5, *p* = 0.001]; simultaneous amplitude discrimination [*r* = 0.24, *r*^2^= 0.06, *F*_(1, 160)_ = 4.86, *p* = 0.03], temporal order judgement [*r* = 0.38, *r*^2^= 0.15, *F*_(1, 160)_ = 13.7, *p* < 0.001], and duration discrimination [*r* = 0.29, *r*^2^= 0.08, *F*_(1, 160)_ = 7.19, *p* = 0.009]. Similarly there were positive linear relationships with MEP:cSP 150% [*r* = 0.21, *r*^2^= 0.05, *F*_(1, 160)_ = 5.81, *p* = 0.02], 170% [*r* = 0.23, *r*^2^= 0.05, *F*_(1, 160)_ = 6.12, *p* = 0.01], and SICI [*r* = 0.18, *r*^2^= 0.03, *F*_(1, 160)_ = 5.37, *p* = 0.02], and LICI [*r* = 0.15, *r*^2^= 0.02, *F*_(1, 160)_ = 4.02, *p* = 0.05].

## Discussion

Here we present novel findings in a large cohort of retired contact sport athletes, specifically that players expressing chronic symptoms showed significantly altered intracortical signalling activity compared to asymptomatic and control groups. Symptom items reflected somatosensory and TMS intracortical inhibition changes, with a positive linear relationship between number of reported concussions and worse outcomes in both somatosensory performance and TMS intracortical inhibition. This study supports the findings of previous smaller TMS studies reporting changes in abnormal GABAergic activity (4, 11–13). Taken together these findings demonstrate that retired athletes who have concerns regarding their cognitive function do exhibit significant underlying pathophysiological changes that would be consistent with persistent structural injury.

Our work has previously utilized the *mental fatigue and related symptom survey* with those suffering with persistent post-concussion symptoms (23, 24). While primarily designed for those with on-going symptoms (25), the instrument has been sufficiently sensitive to detect significant differences between groups in this study. We found that while the “symptomatic” groups were significantly higher than both “asymptomatic” and control groups, we were surprised to observe that the “asymptomatic” was also significantly higher across scores to controls (with the exception of sensitivity, 24-h variation and increased sleep). However, it should be noted that while statistically significant, large effect size differences were not of a magnitude (*d* > 3.0) to suggest the probability that this difference being clinically meaningful (42). Moreover, Johansson and Rönnbäck (43) have proposed that the clinical cut-off score of 10.5, reporting in their samples that a score of 10.5 and above showed significant deviation from controls. Therefore, the mean score of 8.5 in the “asymptomatic” group, while statistically significant, suggests no clinically meaningful difference between these two groups.

One novel component of our study was adding somatosensory measures to our established TMS protocol, which allowed for further study of GABAergic changes in both corticomotor and somatosensory pathways associated with neurological conditions (44–46). Results showed statistically significant differences and large effect sizes between “symptomatic” and control groups across multiple measures where interpretation of tactile stimulation was required (amplitude discrimination, temporal order judgement, and duration discrimination). In the tactile stimulation assessments, there were no statistically significant differences between the “asymptomatic” and control groups, however the moderate to large effect sizes suggest there may be subtle differences that may reflect sub-clinical impairments due to a history of concussion and repeated head trauma more generally. Indeed, the differences in reaction time and reaction time variability between “asymptomatic” and control groups could reflect this sub-clinical change more sensitively than other assessments. Longitudinal studies are required to detect the predictive ability of reaction time and reaction time variability on currently “asymptomatic” retired athletes, and these studies are currently underway.

Collectively, the somatosensory and TMS intracortical inhibition changes in this study demonstrate abnormalities associated with GABAergic activity. Using both techniques individually, in separate studies, we have shown differences in somatosensory responses and TMS inhibition following acute concussion compared to controls (22, 39). More recently however, we have used both techniques conjointly, demonstrating alterations in the GABA system with persistent post-concussion symptoms compared to individuals who had recovered from their concussion (23, 24) with abnormal GABA activity correlated to scores reported in the *mental fatigue and related symptom survey*. Similarly, in the present study, retired players with self-reported ongoing symptoms demonstrated differences in somatosensory responses and TMS intracortical inhibition compared to retired players who did not report any ongoing concerns. Concussion/subconcussion history also revealed positive associations with abnormal somatosensory and intracortical inhibition neurophysiology. While we are not in a position to describe the underlying pathology that may contribute to our pathophysiological findings associated with self-reporting or even concussion/subconcussion history, using TMS and somatosensory techniques allows us to observe deficits in the underlying pathophysiology that play a role in tipping the balance between excitation and inhibition.

Previous experimental work, as models for assessing neurologically impaired patient cohorts, has demonstrated that manipulation of GABAergic pathways can alter TMS inhibition measures and impair tactile perception. For example, pharmacological TMS studies have reported changes in cSP, SICI and LICI reflecting the CNS-active mode of action enhancing (or diminishing) interneuron GABA receptor activity (47). Neuromodulation from repetitive cortical stimulation can also influence GABA activity, and alter sensory processing and perception with Lee et al. (48) demonstrating impaired somatosensory responses following continuous theta-burst stimulation technique to suppress inhibitory networks mediated by GABAergic interneurons. Clinically, Guerriero et al. (49) have posited several mechanisms contributing to alterations in the inhibitory pathways following brain injury that may explain our findings, including deficits in the synthesis of GABA by the glutamic acid decarboxylase, potential gene expression changes influencing GABA activity, or chronic receptor changes due to persistent Ca^++^ influx.

The aim of our study was not only to investigate the neurophysiological differences between “symptomatic” and “asymptomatic” cohorts, but also to provide context in these measures to the individual's self-report of symptoms and concussion history. While increased number of reported concussions showed positive relationships with somatosensory and TMS data, we note that while the somatosensory data correlated with almost all of the self-report measures, TMS measures correlated with just over half of the items. Specifically, TMS showed MEP:SP ratios were negatively associated with overall fatigue and concentration, and sensitivity to stress and irritability, while paired-pulse measures positively correlated to general fatigue, slowness in thinking and memory difficulties. It is interesting to note the different symptom correlations between single and paired pulse measures. As this has been the first time this data has been presented, it would be presumptive to speculate on physiological mechanisms for this observation, and further research is required to ascertain if different symptom items can be detected using TMS, particularly with the inclusion of other paired-pulse paradigms such as intracortical facilitation.

There are limitations in this study which should be noted. Firstly, this study used a mixed sports cohort that not only included various football codes, but also a small number from boxing and car racing. We appreciate that these individuals are exposed to different forms of impacts and therefore potential mechanisms of brain injury, which may have some influence on the results. As previous TMS studies exploring chronic outcomes of repeated head trauma have similarly used mixed cohorts [e.g., (12)], we deem that this has not altered the overall findings significantly. Secondly, we acknowledge that we have utilised a cross-sectional design. Consequently, we were not able to obtain measures of premorbid functioning in the football-playing groups. We have tried to address this however by incorporating an “active-control” group of participants with similar concussion history but no self-reported ongoing concerns. Future research designs should employ repeated measures, if possible, to quantify time-related changes rather than relying on comparisons to a healthy age and education matched control group.

While we used a range of stimulus intensities for MEP:cSP ratio, we only used fixed ISI of 3 and 100 ms intervals for SICI and LICI, respectively. This may have limited our correlation analyses between TMS and somatosensory measures. Future research will aim to incorporate protocols across a range of ISIs including 1–5 ms for SICI, and 100, 150, and 200 ms for LICI, as well as intracortical facilitation with ISI of 10, 12, and 15 ms. The inclusion of more intervals has been used to significantly detect intracortical synaptic and cholinergic circuits impairments that have predicted cognitive decline in Alzheimer's disease (50–52). We intend to draw on this research, utilising a greater number of ISIs in paired-pulse TMS to investigate patients provided with a medical opinion of “probable chronic traumatic encephalopathy” to those of other dementias who have not reported a history of repeated head trauma.

A final limitation is that we did not include a cognitive testing battery. While TMS should be incorporated with “functional” tests (i.e., cognitive or motor assessments) to provide context (44), the aim of this study was to focus on neurophysiological differences between “symptomatic” and “asymptomatic” cohorts. We have previously used cognitive testing batteries (e.g., CogState Brief Battery and Cambridge Neuropsychological Test Automated Battery) to compare retired athletes to age and education matched controls, with mixed results (4, 11). With recent concerns regarding online cognitive testing programs (53), future research will incorporate more comprehensive sensitive and specific neuropsychological evaluations, rather than computerised batteries, alongside TMS and somatosensory testing, that can allow for more refined diagnostics and longitudinal monitoring of future decline as well as management outcomes.

In conclusion, single/paired pulse TMS and somatosensory stimulation provide a low-cost, powerful, multimodal approach to study human brain function. Employing these techniques, this study provides novel neurophysiological evidence of differences in retired contact sport athletes who report ongoing symptoms compared to those with similar concussion histories without ongoing symptoms. Further, there appears to be a dose-response relationship with greater number of concussions in the context of subconcussive exposure showing linear relationships to abnormal neurophysiology. Our research further supports the latest consensus statement that recognises TMS as a viable research technique in the study of concussion (54). Undertaking co-registration studies of TMS and somatosensory stimulation with neuroimaging techniques will provide further understanding of the long-term outcomes of repeated head trauma as well as monitoring techniques for prospective longitudinal studies.

## Data Availability Statement

The raw data supporting the conclusions of this article will be made available by the authors, by reasonable request.

## Ethics Statement

The studies involving human participants were reviewed and approved by La Trobe University HEC18005. The patients/participants provided their written informed consent to participate in this study.

## Author Contributions

AP conceived the study and performed participant testing. BR assisted with participant testing. AP, DK, MT, and AF contributed to manuscript preparation. BR, MB, RM, and JB provided critical review of manuscript drafts and editing. All authors contributed to the article and approved the submitted version.

## Conflict of Interest

AP currently receives partial research salary funding from Sports Health Check charity (Australia) and Erasmus+ strategic partnerships program (2019-1-IE01-KA202-051555). AP has previously received partial research funding from the Australian Football League, Impact Technologies Inc., and Samsung Corporation, and has provided expert reports in concussion legal proceedings. The funders were not involved in the study design, collection, analysis, interpretation of data, the writing of this article or the decision to submit it for publication. MT has received partial funding from the Office of Naval Research. MT was a director of Cortical Metrics LLC who manufactures the Brain Gauge device used in this study. The remaining authors declare that the research was conducted in the absence of any commercial or financial relationships that could be construed as a potential conflict of interest.
